# Herpes Simplex Virus Hepatitis: A Brief Review of an Oft-overlooked Pathology

**DOI:** 10.7759/cureus.4313

**Published:** 2019-03-25

**Authors:** Eric O Then, Vijay Gayam, Vijay S Are, Tagore Sunkara, Vinaya Gaduputi

**Affiliations:** 1 Internal Medicine, St. Barnabas Hospital Health System, Bronx, USA; 2 Internal Medicine, Interfaith Medical Center, Brooklyn, USA; 3 Internal Medicine, Stormont Vail Health System, Lawrence, USA; 4 Internal Medicine, Mercy Medical Center, Des Moines, USA

**Keywords:** viral hepatitis, herpes simplex virus (hsv), liver failure

## Abstract

Herpes simplex virus (HSV) is a rarely reported cause of viral hepatitis. Aggressive in nature, most cases of HSV hepatitis rapidly progress to fulminant hepatic failure. Present day, its pathogenesis is yet to be elucidated, but its complications and associated high mortality rate are clear. Clinically, its symptoms mimic those of other causes of acute hepatic failure thus making the diagnosis of HSV hepatitis a precarious task. Although treatment in the form of acyclovir is readily available, most cases have a poor prognosis due to late initiation of therapy. This makes the early identification of HSV essential in improving outcomes and potentially preventing mortality.

## Introduction and background

Fulminant hepatic failure is defined as severe liver injury resulting in impaired synthetic capacity and encephalopathy in patients with previously normal liver function [1]. It is typically characterized by increases in transaminases, which serves as the first clue to the clinician that acute liver injury is occurring. Depending on how elevated the patient’s transaminases are, one can then narrow the list of differential diagnoses. Elevations in transaminases greater than ten times the upper limit of normal are suggestive of ischemic, toxic or viral liver injury [2]. Of these, toxic (acetaminophen) and viral are the most common etiologies worldwide [3]. Hepatitis A, B, C, D, and E are the most frequently encountered forms of viral hepatitis in the medical literature. Rarely will non-hepatitis viruses cause fulminant hepatic failure. The goal of this review article is to aid clinicians in the early identification of herpes simplex virus (HSV) hepatitis in order to initiate rapid treatment and ultimately prevent mortality.

## Review

Epidemiology

HSV hepatitis in adults is a rare entity that was first reported in 1969 by Flewett et al. [4]. Since its discovery, it is thought to comprise a mere 1% of all cases of acute liver failure (ALF), and only 2% of viral associated ALF [5]. In the limited amount of cases that have been reported in the literature, it has occurred more frequently in patients that are in an immunocompromised state. These states include pregnancy, corticosteroid use, human immunodeficiency virus (HIV), and autoimmune disease [6]. There also seems to be a female predilection of the disease. In 2007, Norvell et al. compiled 137 cases of HSV hepatitis that were available in the literature at the time. Their data demonstrated a female predominance of 62%. In the same study, 76% of inflicted patients were found to be either pregnant or immunosuppressed [7]. It is important note however that HSV hepatitis is non-discriminating and may occur in male immunocompetent patients as well [8].

Pathogenesis

There are multiple theories regarding the pathogenesis of HSV hepatitis. The first theory postulates that a large HSV inoculum at the time of initial infection overwhelms the hosts defense system, and results in visceral dissemination into the liver. The second theory suggests that visceral dissemination derives from herpetic lesions in the setting of impaired macrophages, cytotoxic T-lymphocytes, and delayed type hypersensitivity. The third theory suggests that an acute HSV infection, superimposed on a latent HSV reactivation, may be the culprit in the development of fulminant hepatitis. Finally, a fourth theory takes into consideration the heterogeneity of HSV. Studies have proven that certain strains of HSV are neurovirulent causing herpetic encephalitis. Similarly, there may be certain strains that are hepatovirulent and result in fulminant hepatic failure [9, 10].

Clinical manifestations

Identifying HSV hepatitis can be a precarious task. Clinically its presentation is indistinguishable from other etiologies of acute hepatitis. In addition, many of the cases reported in the literature have presented with an anicteric pattern [11]. To further complicate matters, less than half of the reported cases present with mucocutaneous lesions suggestive of HSV [12]. Leukopenia and thrombocytopenia have also been reported to be associated with HSV hepatitis [13]. Other findings that are often seen in HSV hepatitis include fever, coagulopathy (resulting in life-threatening hemorrhage), and acute renal failure [7]. Given the lack of objective physical findings, and absolute laboratory results, history gathering becomes essential.

Diagnosis

Diagnostic tools that can aid a physician in recognizing HSV hepatitis include HSV serology, HSV DNA by polymerase chain reaction (PCR), computed tomography (CT) scan and liver biopsy. Regarding laboratory studies, HSV DNA by PCR has been shown to be more discriminating than serologic testing for diagnosing or excluding HSV as a cause of ALF [14]. CT scan findings are very non-specific, but may show hepatomegaly along with diffuse hypodense 1-4 mm lesions, which represent foci of acute hepatic necrosis. Ultrasound may also be useful in excluding other diagnoses that are suspected. This is especially true in pregnant patients where fatty liver disease of pregnancy is often the cause of acute liver failure [15]. Despite the utility of these diagnostic modalities, liver biopsy remains the gold standard for diagnosing HSV hepatitis. Histologically, biopsy will show focal or confluent areas of acidophilic type necrosis, with little associated inflammation. In non-necrotic areas, Cowdry type A bodies surrounded by halos, may also be found [16].

Treatment

Treatment of HSV hepatitis, and subsequent prognosis, is highly contingent on time. Rapidly progressive acute liver failure occurs in 74% of cases, with mortality rates reaching 90% [17]. Studies have demonstrated better clinical outcomes in patients who were started on acyclovir early in their hospital course. One literature review in particular, concluded that patients who received early treatment were less likely to die, or require liver transplantation [18]. It is important to note however that there have been cases of acyclovir resistant HSV hepatitis reported in the literature. Resistance of HSV to acyclovir is most often seen in immunocompromised patients and rates have been reported to range from 3.5% to 10% in this population [19]. Acyclovir is a nucleoside analogue that is phosphorylated into its active form by thymidine kinase in HSV-infected cells. Acyclovir resistance results from mutations in the thymidine kinase gene that cause decreased production or complete absence of thymidine kinase [20]. In cases of acyclovir resistant HSV hepatitis, Foscarnet or Cidofovir may be employed. These two agents inhibit the catalytic unit of viral DNA polymerase and do not require activation by thymidine kinase [21]. Chaudhary et al. reported one case of HSV hepatitis that showed no improvement with acyclovir, but when foscarnet was added the patient’s mental status improved and she was ultimately discharged home with no need for liver transplantation [22]. Other important questions to consider with regards to treatment are the optimal route of administration (intravenous vs oral) and the subsequent duration of therapy. This is highlighted by a case reported by Czartoski et al. in which a patient diagnosed with HSV hepatitis initially showed improvement on intravenous acyclovir, but quickly decompensated when switched to oral valacyclovir [23]. Despite completing 43 days of intravenous acyclovir prior to discontinuation, the patient ultimately expired. Once fulminant hepatic failure occurs the only option that remains is liver transplantation. In Norvell et al.’s literature review, seven patients with HSV hepatitis underwent orthotopic liver transplantation after developing fulminant hepatic failure. A mere three patients ultimately survived, expiring due to complications associated with liver transplantation [7]. Despite a lack of evidence, there may be a role for plasmapheresis in HSV hepatitis. Holt et al. reported one such case in which a pregnant patient presented to their institution with acute liver failure. Given the patient's anemia, thrombocytopenia and transaminitis the presumptive diagnosis of HELLP (hemolysis, elevated liver enzymes, low platelet count) syndrome was made. This was followed by the rapid initiation of therapeutic plasma exchange (TPE). One day after the initiation of TPE the patient’s transaminitis markedly improved (Alanine transaminase decreased by 5,131 IU/L; Aspartate transaminase decreased by 1,282 IU/L). The patient was later diagnosed with HSV hepatitis through liver biopsy and was started on acyclovir. She was ultimately discharged home with normal transaminase levels [24].

## Conclusions

In conclusion, HSV hepatitis is a rare cause of rapidly progressive acute hepatitis that oftentimes results in fulminant hepatic failure. Due to its rarity, and lack of discernible clinical features, early recognition and history gathering become indispensable. Current guidelines do not recommend empiric treatment with acyclovir, but given its high mortality rate and complications associated with liver transplantation, one should consider empiric treatment in selected patient populations where there is a high index of clinical suspicion for HSV hepatitis. These would include immunocompromised patients or promiscuous patients with new sexual partners who present with acute liver failure. In these patients empiric treatment should be strongly considered along with early testing to rule out HSV hepatitis.
